# Cell Cycle Regulation by Integrin-Mediated Adhesion

**DOI:** 10.3390/cells11162521

**Published:** 2022-08-14

**Authors:** Siamak A. Kamranvar, Bhavna Rani, Staffan Johansson

**Affiliations:** Department of Medical Biochemistry and Microbiology (IMBIM), Biomedical Center, P.O. Box 582, Uppsala University, 752 36 Uppsala, Sweden

**Keywords:** adhesion, integrin, cell cycle, mitosis, G2 phase

## Abstract

Cell cycle and cell adhesion are two interdependent cellular processes regulating each other, reciprocally, in every cell cycle phase. The cell adhesion to the extracellular matrix (ECM) via integrin receptors triggers signaling pathways required for the cell cycle progression; the passage from the G1 to S phase and the completion of cytokinesis are the best-understood events. Growing evidence, however, suggests more adhesion-dependent regulatory aspects of the cell cycle, particularly during G2 to M transition and early mitosis. Conversely, the cell cycle machinery regulates cell adhesion in manners recently shown driven mainly by cyclin-dependent kinase 1 (CDK1). This review summarizes the recent findings regarding the role of integrin-mediated cell adhesion and its downstream signaling components in regulating the cell cycle, emphasizing the cell cycle progression through the G2 and early M phases. Further investigations are required to raise our knowledge about the molecular mechanisms of crosstalk between cell adhesion and the cell cycle in detail.

## 1. Integrins

**Figure 1:**
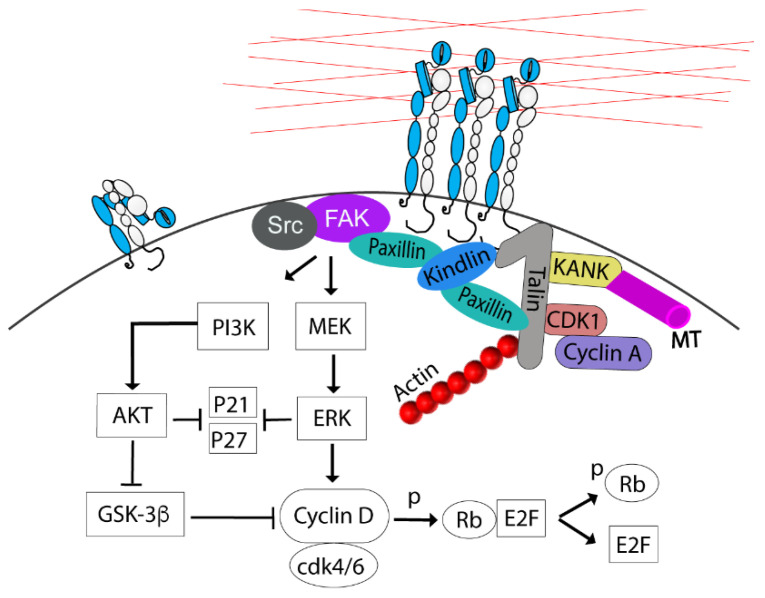
A schematic picture showing some of the main components of CAs, the interaction of cell cycle kinase CDK1 with talin, and the central adhesion-dependent events regulating the G1 to S transition. Paxillin both links kindlin to the GTP-Rap1-activated talin and recruits FAK to the complex. Integrin α-units are shown in blue and the β-units are in white. The red lines represent the extracellular matrix.

Cell adhesion to the ECM is mainly mediated by the integrin family of receptors, which sense the chemical and mechanical properties of the extracellular microenvironment and generate responses regulating many cellular functions, including cell proliferation [1,2]. These heterodimeric receptors, consisting of one of 18 α and one of 8 β subunits, activate signals by ligand-induced clustering of integrins and their associated proteins, as well as by mediating mechanical force to stretch-sensitive proteins. The ability of integrins to bind extracellular ligands with high affinity requires a large conformational shift induced through the interactions with the cytoplasmic proteins talin and kindlin; this occurs when talin is activated by GTP-Rap1 and acts in concert with kindlin via paxillin [3,4,5] (exceptions are integrins αVβ8 and α6β4 [6]). Upon ligand binding, the integrins form different types of adhesion complexes such as hemidesmosomes, reticular adhesions (RA), and canonical adhesions (CAs); the latter is a collective name for the related structures known as nascent focal complexes, focal adhesions (FAs), and fibrillar adhesions, which all are linked to actin and contain the same core components (Figure 1) [7,8,9]. In CAs, the clustering of integrins induces focal adhesion kinase (FAK) trans-autophosphorylation at Tyr-397 [10], a docking site to recruit and activate Src family kinases and PI3 kinase, and this is a starting point for many downstream signaling pathways [11,12]. In addition to activating integrins, talin binds directly to actin filaments and indirectly to the cortical microtubule (MT)-stabilizing complex via the KANK family of proteins [13,14]. The RAs, also known as flat clathrin lattices, contain αVβ5 integrin and components of clathrin-mediated endocytosis but lack FA proteins and F-actin-linking mediators. In cultured cells, RAs are formed during interphase and preserved throughout the cell cycle [7,15]. However, in vivo, RAs appear to have limited distribution, mainly found in skeletal muscles and osteoclasts, and their functions have not yet been extensively explored. Hemidesmosomes contain integrin α6β4 linked to intermediate filaments and are known to generate signals, but their contribution to the cell cycle regulation is still unclear [16].

## 2. Cell Cycle and Cell Adhesion Crosstalk

“Anchorage-dependent growth”, or more correctly, anchorage-dependent proliferation, is a long-known feature of normal mammalian cells that is lost in tumor cells. This dependency applies also to leukocytes, which normally are produced from adherent progenitor cells in the bone marrow and proliferate after extravasation into tissues upon the switching from a non-adhesive to adhesive phenotype. Some stem cells are an exception to the rule and can form colonies from a single cell in suspension [17,18]. The significance of CAs for anchorage-dependent proliferation and cell cycle regulation is extensively documented [19], particularly in the G1 to S phase transition [20] and cytokinesis [21]. More recently, CDK1, one of the cell cycle master kinases, has been found to have critical regulatory roles both for CA assembly during the S phase and disassembly before the mitosis entry [22]. These findings illustrate the close integration of CA signaling with cell cycle regulation. In the following sections, we discuss the molecular mechanisms by which cell adhesion signaling might influence the cell cycle progression at several stages. 

### 2.1. G1 to S Transition

The contribution of integrin signaling to the G1/S transition has previously been reviewed in detail [23]. Briefly, integrin adhesion via FAK activates PI3K/AKT and MAPK/ERK signaling pathways to upregulate cyclin D levels and accelerate the degradation of the CDKs inhibitors (p21, p27), required for the G1/S transition [24,25,26]. ERK stimulates cyclin D1 gene transcription by regulating AP-1, Ets-like factor, and KLF8 transcription factors [23,27,28]. The cyclin D1 protein level is also controlled by proteosome-dependent degradation after marking by GSK-3β-mediated phosphorylation. Activating the PI3K/AKT pathway inhibits GSK-3β, thereby preventing premature cyclin D1 degradation [29]. Cyclin D1 binds and activates CDK4 and 6, which then phosphorylate the retinoblastoma protein (Rb), and thereby the E2F family of transcription factors is released to target genes required for entry into the S phase (Figure 1) [23]. Integrins share similar downstream signaling pathways with growth factor (GF) receptors necessary for passing through the G1/S checkpoint. However, detached non-transformed cells cannot progress into the S phase even in the presence of GF [30], possibly because the more sustained signaling from integrin adhesion is required for achieving sufficient levels of cyclin D1 [20,31].

After the cells have entered the S phase, the CAs expand in size and number, and the actin filament organization is altered from peripheral to cell-traversing in 2D cultures. These morphological changes are driven by CDK1 associated with cyclin A2 [22,32]. Cyclin A2/CDK1 has many phosphorylating targets on the components of the adhesion complex [22,32], including S1589 in the R7 domain of talin [33], a modification that may affect talin’s interactions with several proteins, including KANK, RIAM, and paxillin and, thereby, regulate CA stability and growth [33]. Moreover, cyclin A2/CDK1-mediated phosphorylation of formin-like 2 protein (FMNL2), a nucleation and elongation factor for actin filament assembly, contributes to the CAs growth presumably through mechanostimulation by actinomyosin of talin and possibly other CA proteins (Figure 1) [22,32,33]. Thus, since talin has a central role in the close interdependence between CAs and actin filaments, these described modifications by cyclin A2/CDK1 can regulate both CA size and actin organization in a coordinated manner. 

### 2.2. G2/M Transition

**Figure 2:**
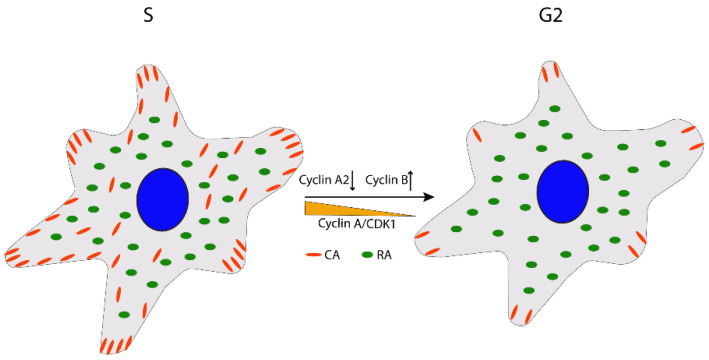
The schematic top view picture indicates that the number and size of CA area is shrinking in the G2 phase mainly due to the inactivation of cyclin A2/CDK1 in the G2 phase while RAs are maintained.

During the G2 phase, the cyclin A2/CDK1 activity declines, leading to shrinkage of the CAs’ area and stress fiber disassembly; possibly, the reduced cyclin A2/CDK1 activity is due to the upregulation of cyclin B expression, which competes for binding to CDK1, and towards the end of G2 the cyclin A2 level also decreases by degradation (Figure 2) [22]. In parallel, at the end of G2, the fine balance between the general CDK1-inhibiting phosphorylation by Wee1 and the CDK1-activating de-phosphorylation by Cdc25c is shifted towards Cdc25c by the increasing PLK1 activity, resulting in rapid activation of cyclin B-CDK1 and mitosis entry [34,35]. Recently, it was discovered that signals from integrin adhesion are involved in the upstream stimulation of PLK1 during the G2 phase, and the absence of FAK expression or its activity caused a reduced level of active PLK1 and a prolonged G2 phase [36]. The cell morphological changes in G2 are correlated with a reduction of traction forces during cell cycle progression from the S phase [37] and are required to prepare the cells for the dramatic rounding at the start of the M phase. Thus, cells will be unable to divide normally if the reduction of adhesion area fails in the G2 phase [38,39].

#### Cell Rounding

**Figure 3:**
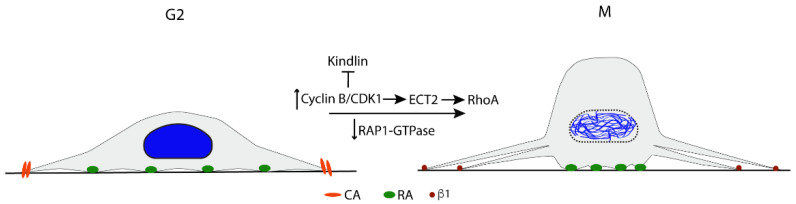
The schematic side view picture indicates cell rounding and the remnants of cell adhesions. The activation of cyclin B/CDK1 and the downstream degradation of kindlin and activation of the RhoA pathway, together with the inactivation of RAP1-GTPase are described as the main mechanisms for cell rounding in mitosis.

The rounding up of the cells from the ECM during prophase to anaphase transition is necessary in order to generate enough 3D space and, thereby, facilitate the capture of all kinetochores by mitotic spindle MTs, as directly demonstrated in experiments where height limits were imposed on cells [40]. Failure in rounding up prolongs the mitotic division time and increases the risk of aneuploidy [38,41]. The cell rounding is driven by increased intracellular osmotic pressure and water influx, together with RhoA-induced contractility of the cortical actomyosin filament network [42,43]. However, a prerequisite for a sufficient effect on the cell shape by these activities is that the CAs have been extensively disassembled into small remnants (Figure 3) [38,44]. 

The mechanism for the CA disassembly at mitosis entry differs from the CA turn-over during interphase, e.g., when cells migrate. One noticeable difference is that cyclin B/CDK1 activation induces CA disassembly and rounding at mitosis, while cyclin B is absent in the G1 phase. Pharmacological inhibition of cyclin B/CDK1 halts the cells at the G2/M interface with flat morphology, and upon washout of the inhibitor, the cells immediately round up [45]. Recently, the cyclin B/CDK1 regulation of CA disassembly was found to depend on its direct phosphorylation of kindlin and the subsequent ubiquitination by Cullin9-FBX10 and degradation. However, a small amount of kindlin remained in a string of puncta together with active β1 integrin, talin, paxillin, and vinculin along the retraction fibers. Among the studied CA-proteins, only kindlin was degraded during mitosis; the others were re-located to the cytoplasm [44]. Notably, β1 integrin was present at the plasma membrane around the cell body and was apparently not internalized, in contrast to the CAs’ turnover by endocytosis during interphase [46,47].

In addition to the degradation of kindlin, several other reactions have been described, which may work in concert and contribute to the mitotic CA disassembly. Rap1 GTPase, a key positive regulator of integrin activity via the promotion of the talin–paxillin–kindlin interaction [3], is rapidly inactivated at the G2/M transition [38,40]. The expression of constitutively active Rap1 inhibits the CA disassembly and the subsequent cell rounding [38,40]. Furthermore, at mitotic entry, FAK undergoes tyrosine dephosphorylation, which results in the loss of both enzyme activity and binding sites for SH2-domain proteins. In addition, FAK serine phosphorylation levels rise concurrently [48]. Similar transient tyrosine dephosphorylation and increased serine phosphorylation also occur in the CA adaptor proteins p130CAS and paxillin [48]. These serine phosphorylations have been suggested to inhibit interactions with CA proteins [48], but further studies are needed to confirm this possibility. Although not well understood yet, however, the dramatic and coordinated shift in the serine and tyrosine phosphorylation patterns of key CA proteins during cell rounding indicates that these events have central roles in the mitotic CA disassembly.

Under common cell culture conditions, i.e., in the presence of serum rich in vitronectin, the rounded cells maintain anchorage mainly via RAs [7,22]. Peripheral RAs were found to be connected to mitotic retraction fibers, just as the small CA remains. Since RAs are formed by integrin αVβ5, a receptor believed to bind primarily to vitronectin and fibronectin, further studies are needed to clarify the role of RAs during mitosis in vivo at different locations where these adhesion proteins are not present, e.g., for cells adhering to laminins or collagen IV in basement membranes.

### 2.3. Bipolar Mitotic Spindle Formation

Although a significant reduction of CA number and size is required for cell rounding, the maintenance of integrin contacts is critical for forming a bipolar mitotic spindle. As mentioned above, at this stage, αVβ5-based reticular adhesions are the most abundant contact type in cultured cells [7], and small β1-containing contacts also remain (Figure 3) [44]. While effects on spindle formation by disturbing β5 functions have not been reported, mutation of integrin β1 in the membrane-proximal NPXY motif of the cytoplasmic domain to NPXA was shown to cause failed spindle assembly and chromosome segregation errors [49]. This mutation, known to inhibit the activation of integrins by talin, also caused a reduced rate of MTs re-growth from centrosomes after nocodazole-induced depolymerization, suggesting that impaired MT polymerization contributed to the spindle defect in the mutant cells [49]. 

We recently described another spindle defect caused by the absence of β1 integrin signals. Non-adherent fibroblasts were found to have a fault in the separation of the two centrosomes to opposing sides of the nucleus and frequently formed a monopolar spindle, while cells re-plated on fibronectin showed a typical bipolar spindle formation [36]. Downregulation of FAK expression in adherent cells, or inhibition of its activity, similarly prevented the centrosome separation. The absence of FAK expression or activity significantly reduced the activating phosphorylation of PLK1 at Thr210 and its indirect target Ser1033 in Eg5 (KIF11), the MT motor protein mainly driving the centrosome separation [50]. These data suggest that integrin adhesion is critical in bipolar spindle formation by regulating PLK1 activity via FAK [36]. The Thr210 phosphorylation of PLK1 is known to be carried out by Aurora A, and additional modifications have been reported to affect further the activity and localization of the PLK1 [51]. The mechanism by which FAK regulates PLK1 during late G2/prometaphase is presently unclear. 

**Figure 4:**
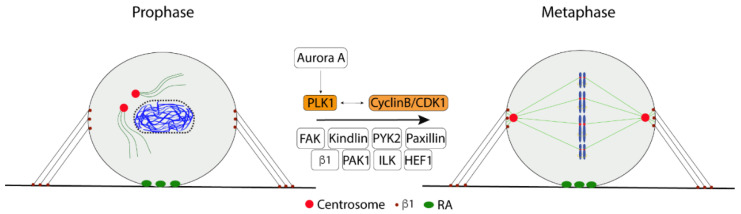
The components of CAs are involved in the cell cycle progression through the G2 phase and early mitosis where cyclin B/CDK1 and PLK1 play critical roles.

Several focal adhesion proteins, including HEF1 (CAS-L), ILK, kindlin, paxillin, PAK1, and PYK2, have been reported to localize at the centrosome during mitosis and to be involved in the spindle organization [52,53,54,55,56,57]. Interestingly, HEF1, ILK, and PAK1 may regulate the spindle via Aurora A or PLK1 [55,56,57] and could thus be possible links between integrin/FAK and Aurora A/PLK1/Eg5 (Figure 4). 

Based on studies where kindlin-1 (epithelial cells) or -2 (neuroblastoma cells) were depleted, both proteins were reported to maintain spindle integrity in human mitotic cells by inhibiting the MT-associated histone deacetylase 6 (HDAC6) [58,59,60]. Since cooperation of kindlin, paxillin, and talin is required for the activation of integrins [3], it appears likely that the spindle defect in the absence of kindlin is due to absent CA and the signals emanating from them rather than other possible kindlin functions. This was also concluded by Patel et al. [59] as similar effects were seen after the knock-down of either talin or kindlin-1. In agreement with this conclusion, depletion of kindlin-2 was shown to reduce the spindle MT acetylation via lack of integrin-induced activation of AKT and, thereby, an increased activity of GSK3β and its downstream target HDAC6 [60]. However, in addition to the role of kindlins in CA formation, kindlin-1 was also found to interact with HDAC6 at MTs close to centrosomes and suggested to regulate α-tubulin acetylation directly [58,59]. The localization at spindle MTs depended on the phosphorylation of kindlin-1 by PLK1 [59]. Notably, in contrast to kindlin-1, kindlin-2 was not found to localize at spindle MTs or centrosomes [60].

Reduced acetylation of α-tubulin during mitosis has been correlated with phosphorylation of FAK at Ser 732 occurring at this stage of the cell cycle. Ser-732 phosphorylated FAK is not found in CAs but colocalizes with the spindle MTs and contributes to MT dynamics by promoting the depolymerization [61]. Thus, in this regard, it appears to act oppositely to kindlin-1. 

### 2.4. Spindle Orientation

The mitotic spindle alignment and positioning play a critical role in cell differentiation, embryogenesis, and organogenesis. The spindle misorientation is reported to cause polycystic kidney disease and is suggested to be involved in the cancer development [62,63]. The cell geometry is critical to determining the spindle orientation. In adherent cultured cells, the spindle axis is coordinated with the longest axis of the cell parallel to the cell–substrate adhesion plane [64]. In cells adhering to fibronectin or collagen, β1-integrins (i.e., CAs) were shown to direct the spindle orientation by accumulating phosphatidylinositol-3,4,5-triphosphate (PtdIns(3,4,5)P3) at the mid-cortex where dynein/dynactin then localizes [64,65]. RAs also regulate the spindle orientation as shown by randomization of the spindle orientation axis in several cell lines after β5 depletion [7].

**Figure 5:**
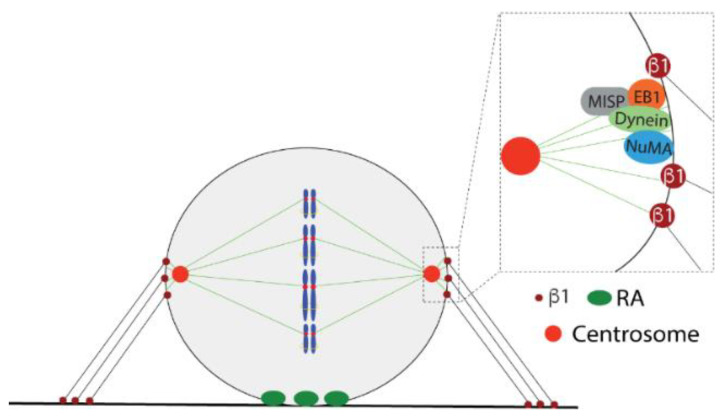
The schematic picture demonstrates the spindle orientation and some of the microtubule- and retraction fiber-associated proteins.

Dynein/dynactin attaches to the cell cortex via NuMA, and the pulling force exerted by this motor protein on astral MTs is the main factor determining the spindle orientation [66,67,68]. The association of astral MTs with the cell cortex and with dynein/dynactin involves several proteins, including EB1and MISP (Figure 5). MISP has also been reported to interact with FAK, and to connect MTs and actin with FAs. MISP is phosphorylated by CDK1 during mitotic entry and re-localizes from FAs to the interface between retraction fibers and the rounded cell body, where it interacts with EB1 and the dynein/dynactin complex [69]. Intriguingly, activated β1 integrin and FAK were also found at this location and to be required for the proper spindle orientation [70].

### 2.5. Chromosome Condensation

The regulator of chromosome condensation 2 (RCC2) is a component of the integrin αVβ3 and α5β1 adhesomes. It is also a key protein in the chromosomal passenger complex (CPC) as the regulator of Aurora B kinase activity involved in chromosome condensation, accurate chromosome segregation, and cytokinesis [71,72]. RCC2 silencing inhibits mitotic spindle assembly leading to activation of the spindle assembly checkpoint and, thereby, mitotic arrest in the prometaphase [73]. Regarding its role in adhesion, RCC2 has been reported to reduce RAC activity and MT stability in CAs and to affect cell migration. RCC2 has guanine exchange factor (GEF) activity for RalA, which promotes the correct localization of the CPC components at centromeres [74,75]. While GTP-RalA potentially may also mediate the effects of RCC2 at integrin adhesion sites, it is presently unclear if there is a connection between the functions of RCC2 at adhesion sites and centromeres.

### 2.6. Cytokinesis

The completion of cytokinesis is well-established as a cell adhesion-dependent stage of the cell cycle [21,76,77], but the underlying mechanism is not yet entirely understood. Cytokinesis starts during anaphase and proceeds sequentially through several stages, including cleavage furrow ingression, midbody formation, and eventually abscission [78]. Integrin-mediated adhesion is essential for the final step of midbody maturation and, thereby, for the abscission process [79,80,81].

Tumor cell lines commonly divide independently of integrin adhesion; thus, their cytokinesis obviously proceeds through mechanisms that differ from non-transformed cells and would be important to understand in detail in order to identify potential tumor-specific treatment targets. Notably, variations in the regulation of the process have also been reported in normal cells. In epithelial cells, cytokinesis was shown to be dependent on the activation of ERK and RSK, whereas this was not the case for fibroblasts [80]. Another example of cell-type-specific variations relates to the role of mechanical tension: dermal fibroblasts failed in cytokinesis more frequently on a soft surface than on stiff surfaces, whereas mesenchymal stem cells divided efficiently independently of the surface stiffness [82]. However, for both fibroblasts and epithelial cells, we found that integrin signaling is involved in cytokinesis through a FAK/PLK1/Cep55 pathway, which is required to recruit ESCRT components TSG101 and Alix to the midbody [21]. According to these results, activated FAK slows down the degradation of PLK1, which regulates the timely localization of Cep55 to the midbody. 

**Figure 6:**
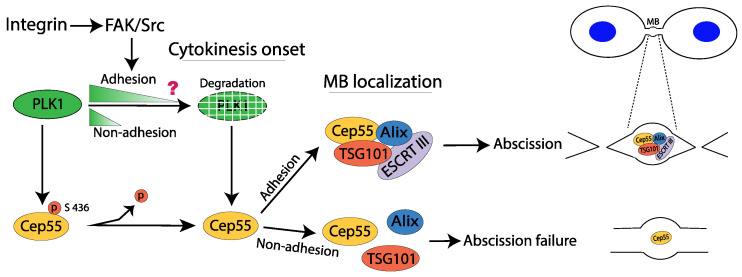
A proposed model for the role of integrin-mediated adhesion signaling via FAK and Src in cytokinesis abscission. Adhesion is indicated to delay the degradation of PLK1 and, thereby, prevent premature recruitment of Cep55 to the midbody; the details of this mechanism are incompletely known. In the absence of adhesion, Alix and TSG101 do not bind to Cep55 and the abscission process cannot proceed further.

PLK1 phosphorylates Cep55 at serin 436 to prevent its localization to the immature midbody in the early cytokinesis, and after PLK1 is gradually degraded, Cep55 gathering at the midbody is permitted. TSG101 and Alix will bind to Cep55 and mediate the subsequent polymerizing of ESCRT-III subunits at the midbody to finalize the abscission process [83]. We found that PLK1 is rapidly degraded in the absence of FAK/Src signaling, which results in premature accumulation of Cep55 at the midbody and subsequent failure in recruiting ESCRTs (Figure 6) [21]. An important question for further studies is why the timing of Cep55 accumulation at the midbody is crucial for the binding of Alix and TSG101.

#### Consequences of Failed Cytokinesis

Cytokinesis failure (due to insufficient adhesion signaling or other causes, such as viral infections, endoreplication, mitotic slippage, and mutations) results in a binuclear cell (4N) with two centrosomes, which after duplication in the S phase causes the formation of multiple spindle poles, chromosome segregation errors, and aberrant mitoses, and all of these events contribute to chromosomal instability and cancer [84,85]. Luckily, in normal cells, cytokinesis failure causes the activation of the p53-dependent cell-cycle arrest, which was previously considered as a “tetraploidy checkpoint” but now is known to be induced by a multiprotein complex containing p53-induced protein with a death domain 1 (PIDD1) [86,87]. Recent studies have shown that the PIDDosome complex is activated when two mature centrosomes merge post cytokinesis failure, and via mother-centriole-associated PIDD1 stabilizes p53 through caspase-2-dependent MDM2 cleavage. Thereby, p53-induced p21 expression halts the proliferation of cells carrying extra centrosomes in the G1 phase of the cell cycle and the cells become senescent [86,87,88]. Although senescent cells can have negative effects on the surrounding microenvironment through the secretion of inflammatory cytokines [89], the PIDDosome activation appears to be a potent anti-tumor mechanism. However, cells with inactivated p53 (due to mutations or viruses) escape the PIDDosome block, and when reaching the next mitosis with >2 centrosomes, such cells must cluster them into two polar groups to form a pseudo-bipolar mitotic spindle to avoid cell death by mitotic catastrophe. But a pseudo-bipolar spindle increases the frequency of lagging chromosomes in anaphase and thus explains the link between cytokinesis failure, supernumerary centrosomes, chromosomal instability, the start of a neoplastic state, and progression to malignant stages [85].

## 3. Future Perspectives

The cell cycle is regulated by the combined effect of several types of stimuli in addition to adhesion signals, including cytokines, metabolites, energy status, and reactive oxygen species [90,91]. Here, we have focused only on the crosstalk between signals to and from integrin-based adhesions and the reactions regulating the cell cycle, which in itself is a broad research area of fundamental importance for understanding cell behavior. Recent work has provided significant progress on several topics in this field that has generated and highlighted many new questions for further studies. In the previous sections, we have pointed out some of these questions, and two of them are: How do integrin signals contribute to the regulation of (1) centrosome function and (2) cytokinesis? The separation of two centrosomes for forming a bipolar mitotic spindle requires FAK-dependent activation of the cell cycle master kinase PLK1, but the intermediate steps need to be clarified. Similarly, the regulatory mechanism of cytokinetic abscission by FAK and PLK1 remains to be identified. These questions are of particular interest for tumor biology since proper centrosome function and cytokinesis are crucial for maintaining genome integrity.

## Data Availability

Not applicable.
